# Supplementary material for: HIF-1α-Dependent Metabolic Reprogramming, Oxidative Stress, and Bioenergetic Dysfunction in SARS-CoV-2-Infected Hamsters
Source: Int J Mol Sci. 2022 Dec 29;24(1):558. doi: 10.3390/ijms24010558 (PMC9820273; doi:10.3390/ijms24010558)
Supplement: Supplementary file 1 [file ijms-24-00558-s001.zip › Supplementary material.pdf]

Supplemental Results:

Supplemental Table S4

Signaling pathways altered by SARS-CoV-2 in hamsters. KEGG pathways shown were observed from using the list of proteins with up or downward relative abundance having a P-value < 0.05 and $\geq$ 3 proteins. Protein abbreviations correspond to UniProt gene names.

**Up-regulated Protein Pathways by SARS-CoV-2**	**Proteins**	**P-value**
Complement and coagulation cascades	VTN, FGA, FGG, KNG1	8.8E-6
Platelet activation	FGA, FGG, GNAI2	9.2E-4
Pathways in cancer	GSTM3, KNG1, GNAI2	0.048
**Down-regulated Protein Pathways by SARS-CoV-2**	**Proteins**	**P-value**
Alzheimer’s disease	PSMA2, GAPDH, SLC25A4, PSMA7, RTN4	2.0E-4
Parkinson’s disease	HSPA5, PSMA2, SLC25A4, PSMA7	5.0E-4
Prion disease	HSPA5, PSMA2, SLC25A4, PSMA7	7.0E-4
Spinocerebellar ataxia	PSMA2, SLC25A4, PSMA7	0.001
Pathways of neurodegeneration	HSPA5, PSMA2, SLC25A4, PSMA7	0.005
Huntington’s disease	PSMA2, SLC25A4, PSMA7	0.011
Amyotrophic lateral sclerosis	HSPA5, PSMA2, PSMA7	0.017

Supplemental Figures:

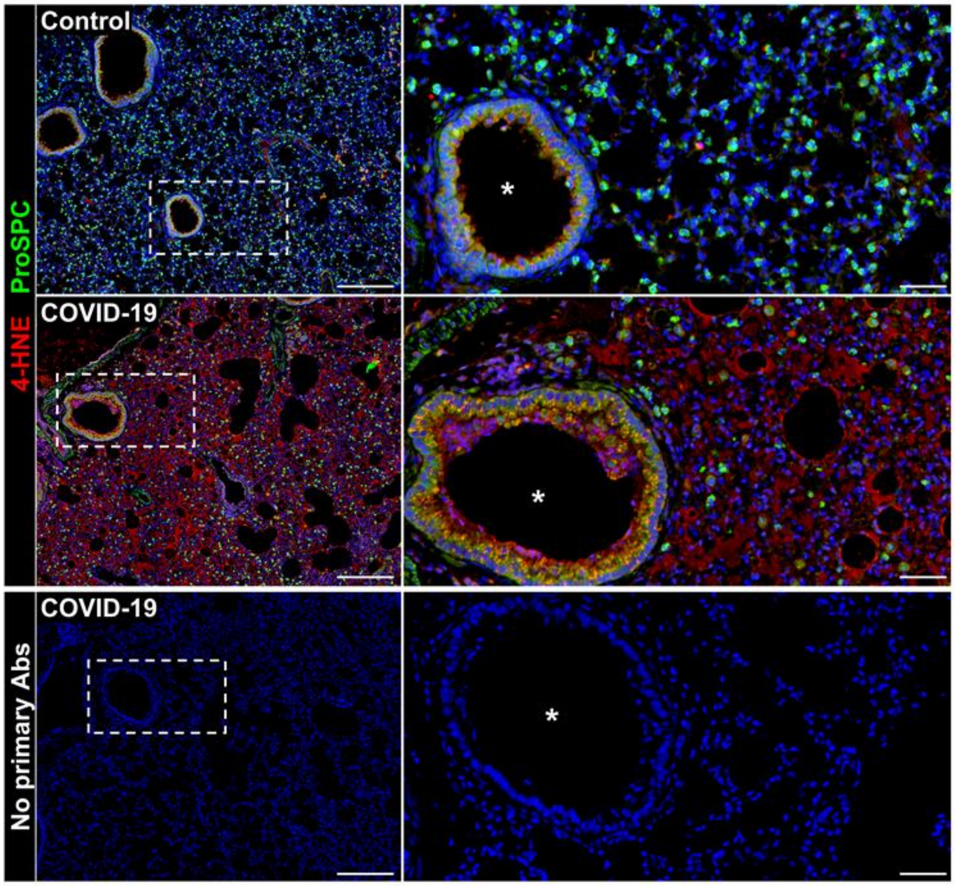

Supplemental Figure S1. Increased 4-HNE deposition in SARS-CoV-2-infected lungs. Double immunofluorescence imaging of 4-HNE (end-product of lipid peroxidation) and ProSPC in lung sections from an uninfected hamster (control) and a SARS-CoV-2-infected hamster at 4 dpi. Control sections show a normal distribution of ProSPC-positive AT2 cells and minimal 4-HNE staining. Infected lungs show extensive 4-HNE deposition in damaged epithelial areas with loss of ProSPC-expressing AT2 cells and in the cellular debris found along the inner lining of bronchioles. No primary antibody experiments showed negative staining in the same infected lung section. Scale bars: 250 µm, 10 µm (magnified panels); * denotes the lumen of a bronchiole.

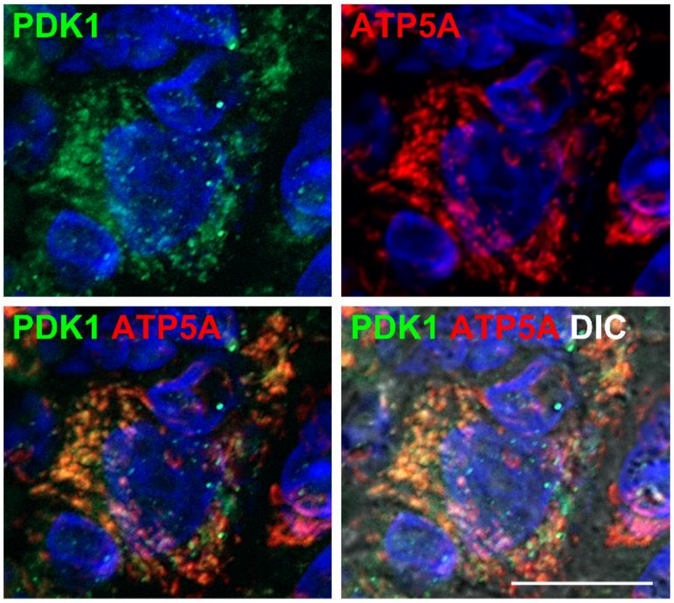

Supplemental Figure S2. Mitochondrial localization of PDK1. Double immunofluorescence imaging of PDK1 and the mitochondrial marker ATP5A in lung sections from a SARS-CoV-2-infected hamster at 4 dpi. Nuclei were counterstained with Hoechst 33342 (blue). Scale bars: 10 µm. DIC = differential interference contrast.

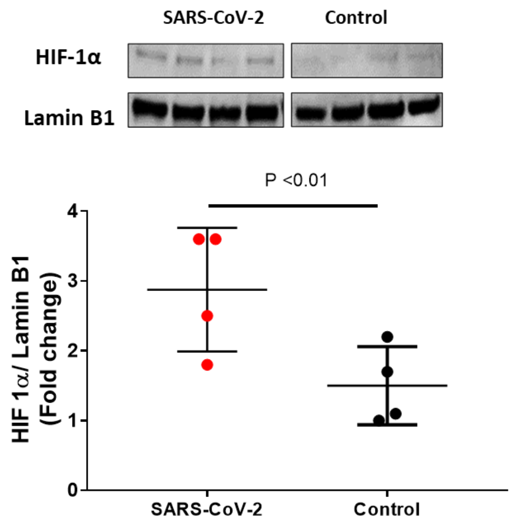

Supplemental Figure S3. Densitometric analysis for relative expression of HIF-1α. Immunoblotting showing HIF-1α expression in nuclear extracts from lung tissue lysates of SARS-CoV-2 infected and non-infected hamsters (upper panel) and Lamin B1 as loading control (lower panel). Densitometry was done by BioRad Image Lab 5.2.1 software.

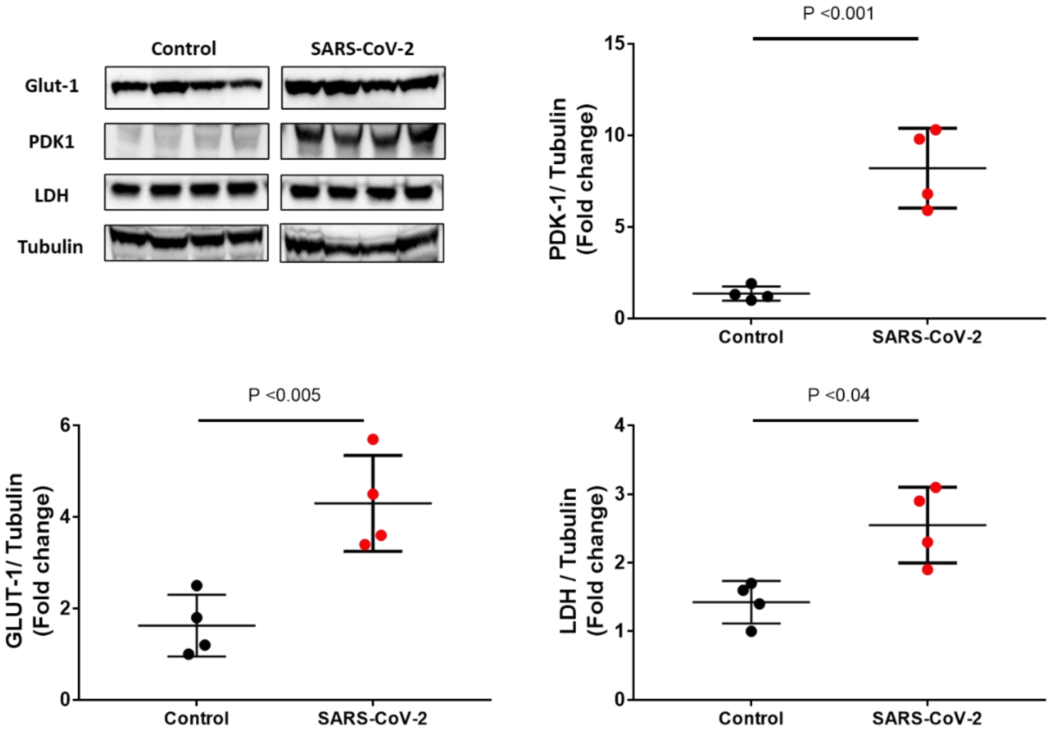

Supplemental Figure S4. Densitometric analyses for relative expressions of Glut-1, PDK1 and LDH. Immunoblotting showing Glut-1, PDK1 and LDH levels in total lung homogenates of SARS-CoV-2 infected and non-infected hamsters. Equal protein loading was confirmed by probing with anti-tubulin antibody (lower panel). Densitometric analyses for relative expression of Glut-1, PDK1, LDH were done by BioRad Image Lab 5.2.1 software.
